# Supplementary material for: Optimization Conditions to Obtain Cationic Polyacrylamide Emulsion Copolymers with Desired Cationic Degree for Different Wastewater Treatments
Source: Polymers (Basel). 2023 Jun 15;15(12):2693. doi: 10.3390/polym15122693 (PMC10303386; doi:10.3390/polym15122693)
Supplement: Supplementary file 1 [file polymers-15-02693-s001.zip › polymers-2426349-supplementary-done.pdf]

polymers

MDPI

*Supporting Information*

# Optimization Conditions to Obtain Cationic Polyacrylamide Emulsion Copolymers with Desired Cationic Degree for Different Wastewater Treatments

**Tung Huy Nguyen [1,*], Linh Pham Duy Nguyen [1,*], Thao Thi Phuong Nguyen [1], Minh Xuan Anh Le [2], Linh Thi Thuy Kieu [3], Huong Thi To [4] and Thanh Tien Bui [1,*]**

[1] Center for Polymer Composite and Paper, School of Chemical Engineering, Hanoi University of Science and Technology, Hai Ba Trung District, Hanoi 11600, Vietnam; nguyenthiphuong@atpcorporation.com.vn
[2] Department of Pharmaceutical Chemistry and Pesticides Tech, School of Chemical Engineering, Hanoi University of Science and Technology, Hai Ba Trung District, Hanoi 11600, Vietnam; lab01@atpcorporation.com.vn
[3] Department of Chemical Process Equipment, School of Chemical Engineering, Hanoi University of Science and Technology, Hai Ba Trung District, Hanoi 11600, Vietnam; linh.kieuthithuy@atpcorporation.com.vn
[4] Lab of Petrochemical Refinering & Catalysis, School of Chemical Engineering, Hanoi University of Science and Technology, Hai Ba Trung District, Hanoi 11600, Vietnam; huong.tothi@atpcorporation.com.vn
* Correspondence: tung.nguyenhuy@hust.edu.vn (T.H.N.); linh.nguyenphamduy@hust.edu.vn (L.P.D.N.); thanhbt.buitien@gmail.com (T.T.B.)

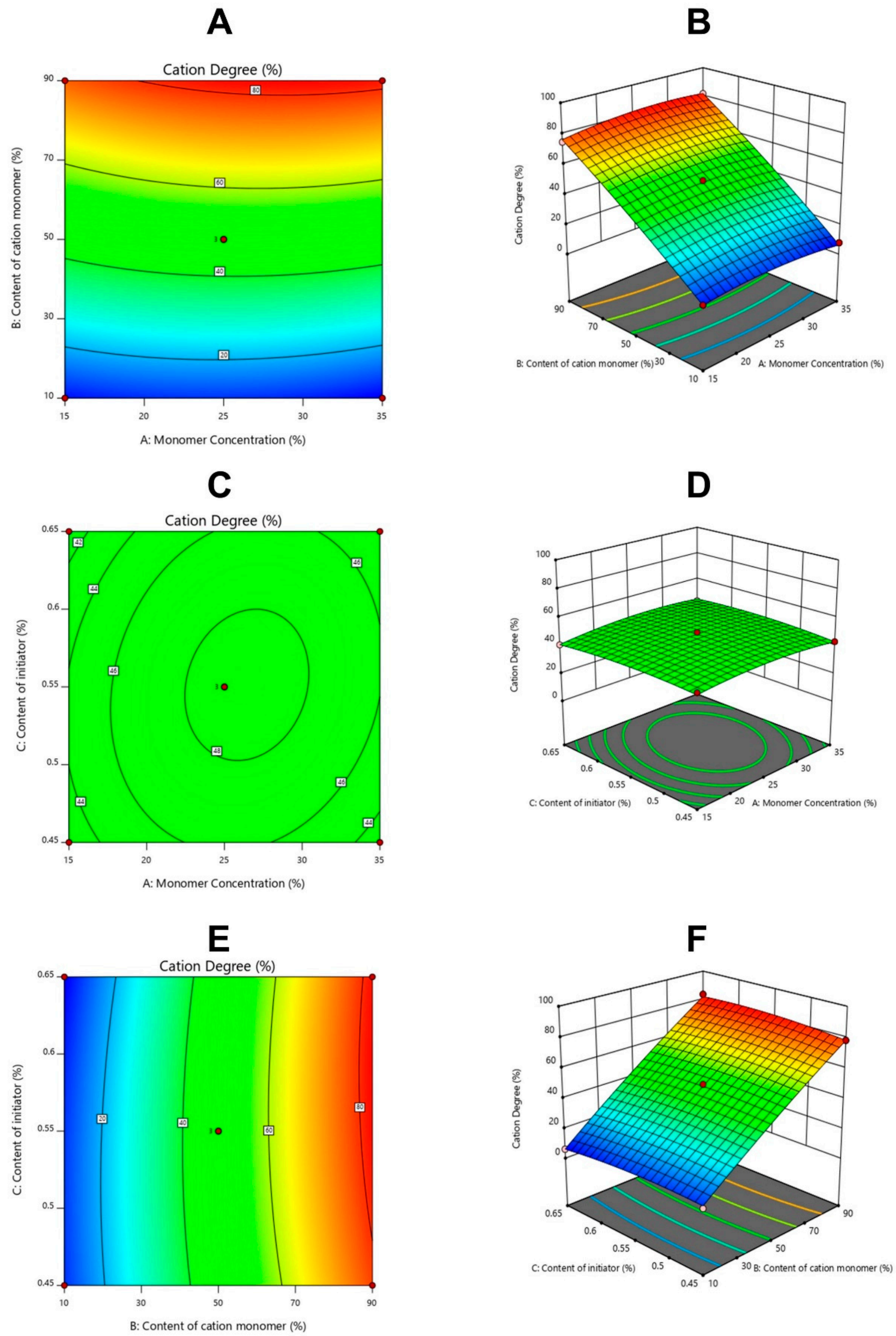


**Figure S1.** 2D contour graphs and 3D response surface: analysis of the interaction effects of the monomer concentration and content of cation monomer; monomer concentration and content of initiator; and content of cation monomer and content of initiator on Cationic degree.

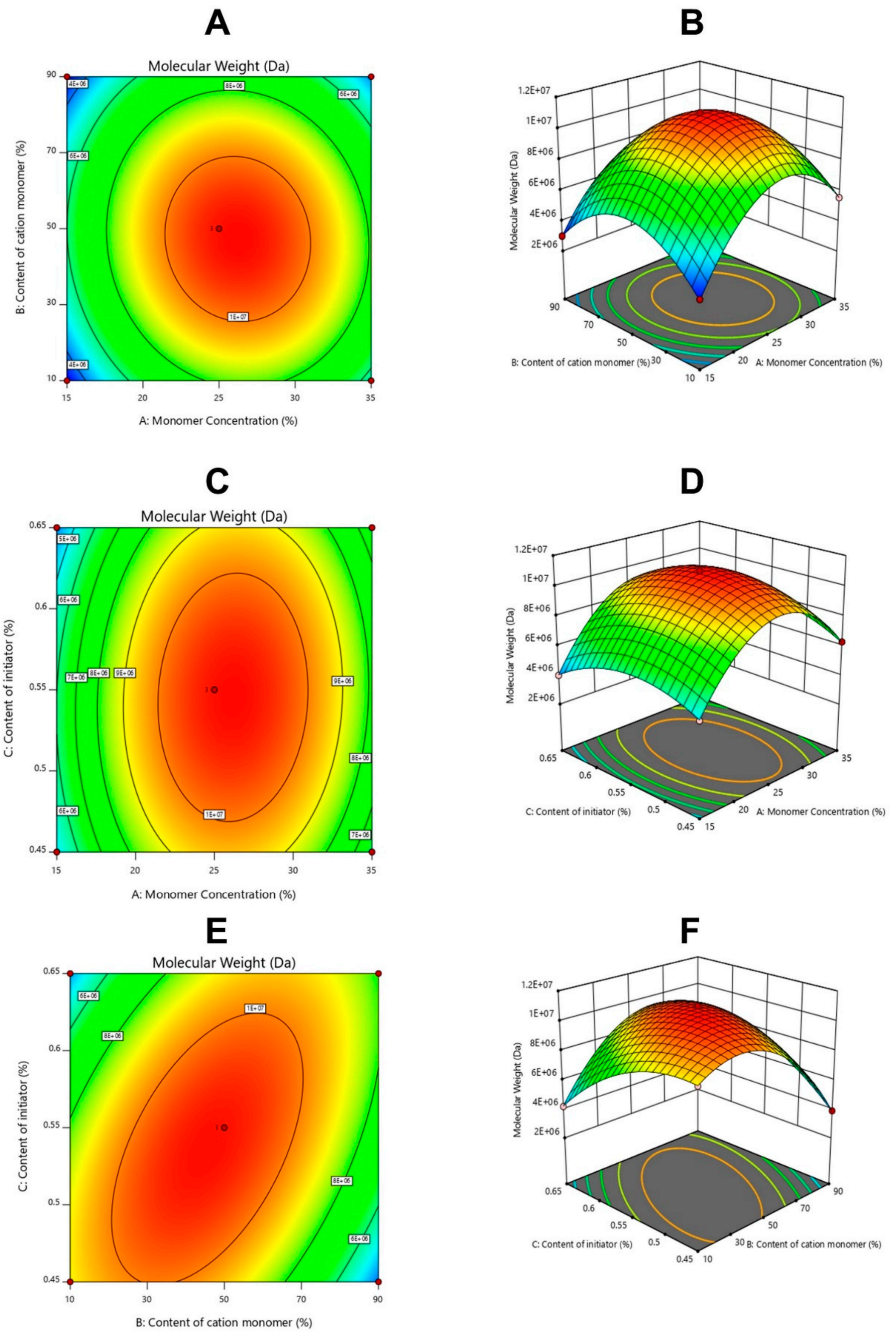


**Figure S2.** 2D contour graphs and 3D response surface: analysis of the interaction effects of the monomer concentration and content of cation monomer; monomer concentration and content of initiator; and content of cation monomer and content of initiator on Molecular Weight.

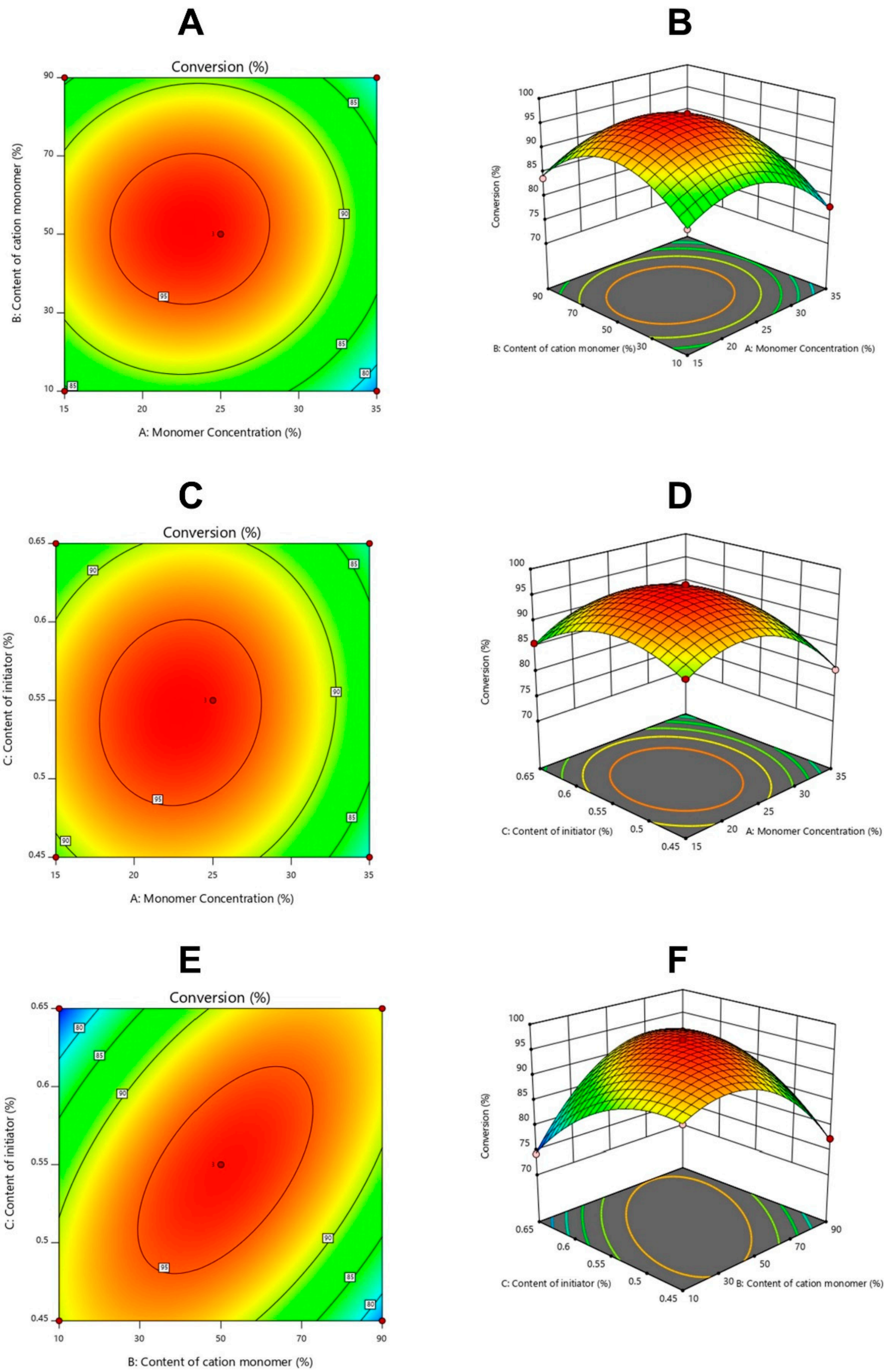


**Figure S3.** 2D contour graphs and 3D response surface: analysis of the interaction effects of the monomer concentration and content of cation monomer; monomer concentration and content of initiator; and content of cation monomer and content of initiator on Conversion.

**Table S1.** ANOVA for the response surface quadratic model for the Cationic degree, the molecular weight, and conversion of CPAM (Cor Total: Corrected Total Sum of Squares).

| Response | Source of variation | Sum of squares | Mean squares | Df | F- value | *p*-value | |
|---|---|---|---|---|---|---|---|
| Cationic Degree | Model | 10485.49 | 1165.05 | 9 | 343.93 | <0.0001 | significant |
| | Residual | 16.94 | 3.39 | 5 | | | |
| | Lack of fit | 14.15 | 4.72 | 3 | 3.38 | 0.2367 | Not significant |
| | Pure error | 2.79 | 1.40 | 2 | | | |
| | Total | 10502.43 | | 14 | | | |
| Molecular Weight | Model | $1.165 \times 10^{14}$ | $1.294 \times 10^{13}$ | 9 | 795.93 | <0.0001 | Significant |
| | Residual | $8.13 \times 10^{10}$ | $1.626 \times 10^{10}$ | 5 | | | |
| | Lack of fit | $2.609 \times 10^{10}$ | $8.698 \times 10^{10}$ | 3 | 0.3151 | 0.8181 | Not significant |
| | Pure error | $5.520 \times 10^{10}$ | $2.760 \times 10^{10}$ | 2 | | | |
| | Total | $1.166 \times 10^{14}$ | | 14 | | | |
| Conversion | Model | 752.74 | 83.64 | 9 | 239.05 | <0.0001 | Significant |
| | Residual | 1.75 | 0.3499 | 5 | | | |
| | Lack of fit | 1.33 | 0.4417 | 3 | 2.08 | 0.3407 | Not significant |
| | Pure error | 0.4243 | 0.2121 | 2 | | | |
| | Total | 754.49 | | 14 | | | |
